# Cancer brings forward oviposition in the fly *Drosophila melanogaster*

**DOI:** 10.1002/ece3.2571

**Published:** 2016-12-20

**Authors:** Audrey Arnal, Camille Jacqueline, Beata Ujvari, Lucas Leger, Céline Moreno, Dominique Faugere, Aurélie Tasiemski, Céline Boidin‐Wichlacz, Dorothée Misse, François Renaud, Jacques Montagne, Andreu Casali, Benjamin Roche, Frédéric Mery, Frédéric Thomas

**Affiliations:** ^1^ CREECMIVEGECUMR IRD/CNRS/UM 5290911 Avenue Agropolis, BP 6450134394Montpellier Cedex 5France; ^2^ Centre for Integrative EcologySchool of Life and Environmental SciencesDeakin UniversityWaurn PondsVic.Australia; ^3^ Laboratoire Évolution, Génomes, et SpéciationUnité Propre de Recherche 9034Centre National de la Recherche Scientifique, 91198 Gif sur Yvette, France; Université Paris‐Sud 1191405OrsayFrance; ^4^ University of LilleCNRSUMR 8198 ‐ Evo‐Eco‐Paleo59000LilleFrance; ^5^ Institute for Integrative Biology of the Cell (I2BC)CNRSUniversité Paris‐SudCEA, UMR919891190Gif‐sur‐YvetteFrance; ^6^ Institute for Research in Biomedicine (IRB Barcelona)BarcelonaSpain; ^7^ International Center for Mathematical and Computational Modelling of Complex Systems (UMI IRD/UPMC UMMISCO)32 Avenue Henri Varagnat93143Bondy CedexFrance

**Keywords:** cancer, fecundity, life‐history strategy, reproduction

## Abstract

Hosts often accelerate their reproductive effort in response to a parasitic infection, especially when their chances of future reproduction decrease with time from the onset of the infection. Because malignancies usually reduce survival, and hence potentially the fitness, it is expected that hosts with early cancer could have evolved to adjust their life‐history traits to maximize their immediate reproductive effort. Despite the potential importance of these plastic responses, little attention has been devoted to explore how cancers influence animal reproduction. Here, we use an experimental setup, a colony of genetically modified flies *Drosophila melanogaster* which develop colorectal cancer in the anterior gut, to show the role of cancer in altering life‐history traits. Specifically, we tested whether females adapt their reproductive strategy in response to harboring cancer. We found that flies with cancer reached the peak period of oviposition significantly earlier (i.e., 2 days) than healthy ones, while no difference in the length and extent of the fecundity peak was observed between the two groups of flies. Such compensatory responses to overcome the fitness‐limiting effect of cancer could explain the persistence of inherited cancer‐causing mutant alleles in the wild.

## 1 Introduction

Life‐history theory predicts that when organisms, both plants and animals, experience environmental conditions that reduce their long‐term reproductive success, a potential way to alleviate costs on their fitness is to invest more than expected in immediate reproductive events, through phenotypic plasticity (Ford & Seigel, 1989; Hirshfield & Tinkle, 1975). Similarly, hosts unable to resist infection by other means (e.g., immunological resistance or inducible defenses) are theoretically favored by selection if they partly compensate for reduced reproductive success due to the parasite by reproducing earlier (Forbes, 1993; Hochberg, Michalakis, & de Meeus, 1992). In this sense, sick individuals may increase their reproductive effort before dying or being castrated by parasites (Adamo, 1999; Minchella & Loverde, 1981; Polak & Starmer, 1998; Vézilier, Nicot, Gandon, & Rivero, 2015).

Cancer, a disease associated with clonal evolution and cell competition within the body, has appeared with the transition to metazoan life (Aktipis & Nesse, 2013). Therefore, dealing with cancer and its fitness‐limiting effect is an ongoing challenge for most, if not all, multicellular species. Despite important differences between infectious diseases and cancers, the impact of tumor development can be closely compared to that of infections by foreign organisms as neoplasia (i.e., new uncontrolled growth of cells that is not under physiologic control) broadly mimics their health as well as their fitness consequences (Vittecoq et al., 2015). Cancer, both solid and liquid tumors, can indeed be seen as an emerging species that behaves in a manner akin to parasites (Duesberg, Mandrioli, McCormack, & Nicholson, 2011). Although cancer is not transmitted, their evolution is governed by the same rules that apply to parasites: cancer cells depend on their hosts for sustenance, proliferating inside their bodies, exploiting them for energy and resources and thereby impairing their health and vigor. In the wild, due to interspecific interactions (especially predation and parasitism), death resulting from cancer per se is likely to occur prior to the end of the reproductive period (Vittecoq et al., 2015). Based on these ecological similarities with infectious diseases, we may predict that the responses evolved in the context of host–parasite interactions should also be relevant in the context of cancer. Although this hypothesis has been proposed (Boddy, Kokko, Breden, Wilkinson, & Aktipis, 2015; Crespi & Summers, 2006), to our knowledge, the adjustment of life‐history traits in order to compensate for the negative fitness costs exerted by oncogenic processes has never been studied (Ujvari et al., 2016).

Using genetically modified fruit flies (*Drosophila melanogaster*) which develop malignant colorectal tumors, we explored the hypothesis of fecundity compensatory responses during the early stages of carcinogenesis. Knowing that APC‐Ras flies display a reduced survival (~50% survival rate 4 weeks after cancer induction compared to ~70% survival rate for healthy flies; Martorell et al., 2014), we specifically tested whether females harboring tumors oviposit more and/or earlier than healthy ones before dying. Knowing that *D. melanogaster* has a fecundity peak early in life (Klepsatel et al., 2013), we expected three possible outcomes of female fly reproduction in response to cancer: (1) an earlier fecundity peak, (2) higher fecundity peak, or (3) a longer fecundity peak.

## 2 Materials and Methods

### 2.1 Biological model

The genetically modified flies *Drosophila melanogaster* used in this study develop a colorectal cancer (CRC) of the anterior gut part. CRC starts with mutations in adenomatous polyposis coli (*Apc*) gene which encodes a large multidomain protein that plays an integral role in intercellular adhesion and in the wnt‐signaling pathway (Nusse & Varmus, 2012). Deregulation of these processes causes unwanted cell growth which may induce tumor development (Fearnhead, Britton, & Bodmer, 2001; Logan & Nusse, 2004; Nusse & Varmus, 2012). The mutations in *Apc* gene are followed by the oncogenic activation of *K‐Ras* gene (involved in the synthesis of protein component of the epidermal growth factor receptor signaling cascade), an event that correlates with the onset of malignancy (Karapetis et al., 2008; Ohnishil, Tomital, Monden, Yana, & Shimanol, 1997). For this research project, we used drosophila clones that combined the loss of *Apc* with the expression of the oncogenic form of Ras. These compound Apc‐Ras clones, generated by a 1‐hr heat shock at 37°C of 2‐ to 4‐day‐old drosophila males and females, expand as aggressive intestinal tumor‐like overgrowths that reproduce many hallmarks of human CRC (Martorell et al., 2014; Wang et al., 2013). Previous experiments performed by us in routine in the laboratory indicated that 100% of the Apc‐Ras clones experiencing a heat shock subsequently develop a colorectal cancer.

### 2.2 Genotypes

yw UAS flp; esg Gal4 UAS‐GFP/CyO; FRT82B Gal80/TM6b flies were crossed with yw hsp70‐flp;UAS‐RasV12/CyO; FRT82B/TM6b flies to generate healthy clones (healthy flies henceforth). yw UAS flp; esg Gal4 UAS‐GFP/CyO; FRT82B Gal80/TM6b flies were crossed with yw hsp70‐flp;UAS‐RasV12/CyO; FRT82B Apc2N175KApcQ8/TM6b flies to generate Apc‐Ras clones (Apc‐Ras flies henceforth). Apc2N175K is a loss‐of‐function allele, ApcQ8 is a null allele, and UAS‐RasV12 is a gain‐of‐function transgene. Flies were sex‐separated at emergence, and MARCM clones were generated by a 1‐hr heat shock at 37°C of 3‐day‐old Apc‐Ras females and were marked by the progenitor cell marker escargot (esg) Gal4 line driving the expression of UAS‐GFP.

### 2.3 Experiment

Ten 3‐day‐old Apc‐Ras virgin females or healthy virgin females were transferred into a small plastic cage (~540 cm^3^) with 10 healthy males. Two petri dishes containing fresh food (per liter of water: 10 g agar, 20 g sugar, and a drop of yeast was added when the medium is solidified)were fixed at the bottom of each cage. Petri dishes were replaced daily by fresh ones, and eggs were counted. Each treatment was replicated 36 times, and we followed the egg production over 2 weeks keeping the flies at 22°C (light/dark cycle 12 hr:12 hr). Dead flies (males and females) were also counted and removed daily to calculate the mean number of eggs laid by females. Males were replaced every 5–7 days by young males to keep sperm production constant.

Knowing that Apc‐Ras and healthy flies are slightly genetically different, we controlled the effect of genetic background by repeating the same experiment, described above, without performing the heat shock (therefore not inducing cancer in the flies with Apc‐Ras mutation). We repeated these two treatments 40 times each at 22°C and counted eggs laying over 7 days.

### 2.4 Statistical analyses

Because our data violate the Gaussian assumption, we have adopted a nonparametric approach where (1) the average age at fecundity peak, (2) the average duration of the fecundity peak, and (3) the average clutch size (or number of eggs) at the fecundity peak were tested through a one‐sided Mann–Whitney test. The duration of the fecundity peak was determined by identifying the last day when the slope of the linear regression between number of eggs laid and duration from the date of fecundity peak become significantly different from 0, that is, when the coefficient associated to the age from the linear regression is significant.

## 3 Results

**Figure 1:**
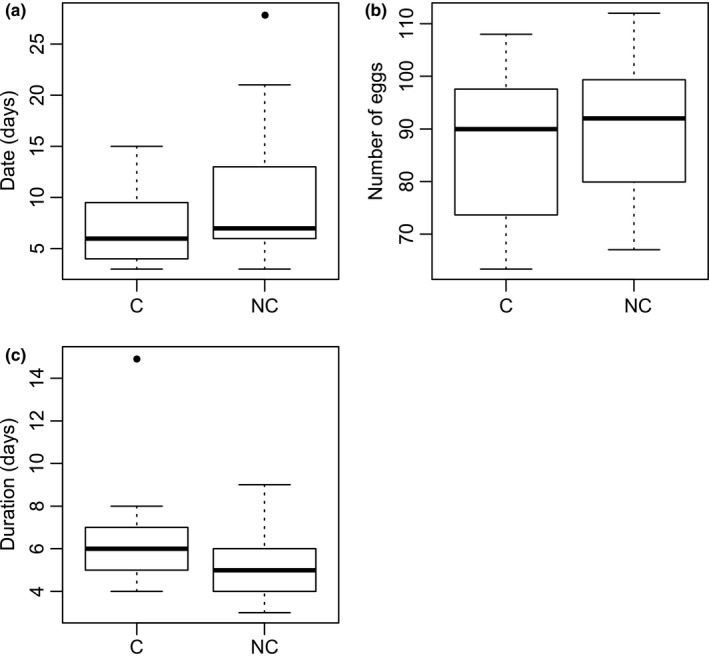
Distribution of the different characteristics of the fecundity peak. (a) Date of fecundity peak (in number of days since the beginning of experiment). (b) The number of eggs at the fecundity peak (in number of eggs). (c) Duration of the fecundity peak (in number of days, see main text for a detailed explanation of this calculation). Only the date of fecundity peak is significantly different between cancerous (c) and noncancerous (NC) individuals (*p*‐value = .025)

**Figure 2:**
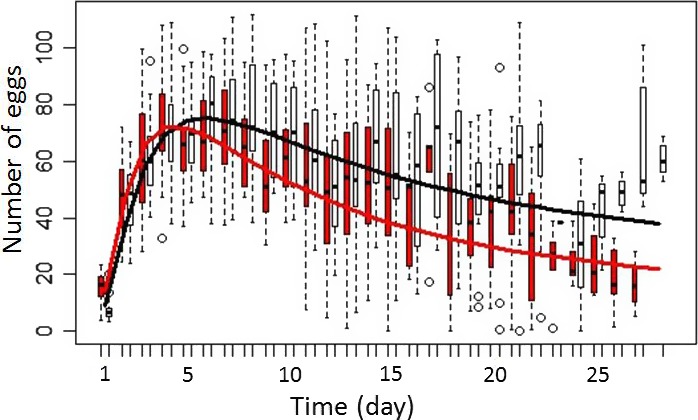
Difference in number of egg laid by cancerous females (in red) and noncancerous ones (in black) as a function of time. The fitted lines are the result of the function: log(Age)*CancerousStatus+log(Age)^2^

We found that fecundity peak occurs at 6.90 days (IC 95%: 5.62–8.18) and at 9.78 days (IC 95%: 7.50–12.06) for Apc‐Ras and healthy flies, respectively (*W* = 305.6, *p*‐value = .025; Figures 1a and 2). However, the total number of eggs at the fecundity peak was not significantly different between Apc‐Ras and healthy flies (86.32, 81.46–91.18, vs. 90.79, 86.15–95.43, eggs, respectively, *W* = 345, *p*‐value = .089; Figure 1b), neither was the duration of the fecundity peak (6.44, 5.28–7.60, vs. 5.27, 4.00–6.54, days, respectively, *W* = 133.5, *p*‐value = .059; Figure 1c).

Considering the experiment without any heat shock (to control the effect of genetic background), there was no difference (*W* = 699.5, *p*‐value = .8538) for the number of eggs laid between the Apc‐Ras (101.47, 92.45–110.54) and healthy flies (95.51, 90.61–100.42) neither for the fecundity peak (4.33 days, 3.99–4.67, for Apc‐Ras and 4.40 days, 4.05–4.75, for healthy flies, *W* = 564.5, *p*‐value = .2848). The duration of the fecundity peak has not been tested because only the first 8 days have been recorded, which is not enough to identify when the linear regression is not significant anymore.

## 4 Discussion

Responses displayed by animals in the face of cancer risks and/or malignant progression have received until now little attention (Vittecoq et al., 2015). The primary reasons this topic has been poorly investigated are because it is often assumed that (1) cancer in wildlife is rare and (2) adaptive responses against cancer are unlikely to evolve because cancer is a postreproductive disease. However, evidence increasingly indicates that these assumptions are wrong, cancer being for instance able to increase the risk that animals die early in life due to predation or parasitism (Martineau et al., 2002; McAloose & Newton, 2009; Roche, Møller, De Gregori & Thomas, in press). Thus, natural selection should favor adaptations that prevent cancer‐induced reductions in fitness, just as we would expect for any other causes (Bernatchez, 2016). Adjustment of life history to compensate for cancer‐induced fitness reduction has not been previously studied (Ujvari et al., 2016).

Unless our finding is due to a collateral consequence of oncogene's activations (e.g., that would concomitantly accelerate egg maturation), our result is in accordance with some of the theoretical expectations that cancer pathologies, as infectious ones, can trigger plastic life‐history adjustments in animals before becoming lethal. Ujvari et al. (2016) indeed predicted that individuals harboring tumors may, as a compensatory response, increase their reproductive effort before dying or being castrated by malignant progression. Here, we found that tumor‐bearing individuals, compared to healthy ones, significantly reached the fecundity peak earlier. Although marginally not significant, we also found that tumor‐bearing individuals tend to lay less eggs than healthy ones during the peak, but tend to display in counterpart a longer peak. These results together could suggest that tumor‐bearing individuals compensate cancer fitness reduction by making an anticipated and slightly longer reproductive effort, even if, presumably because of the pathology, it cannot reach the same level than the one observed with healthy individuals during the peak. Further studies would be necessary to determine how widespread this possible compensatory response among metazoan species is. Importantly, the shift in life‐history traits to counter the fitness‐limiting effect of cancer could potentially explain the persistence of oncogenes in populations. Indeed, although most cancer‐causing mutant alleles are somatically acquired during lifetime (thereafter SCMA for somatic cancer‐causing mutant allele), certain cancers are caused by congenital mutations, that is, germinally inherited cancer‐causing mutant alleles (ICMA) (Arnal et al., 2016). The persistence of ICMA at a higher frequency than expected by the mutation selection balance is, similar to seemingly maladaptive genes, a challenging question for evolutionary biologists (Martineau et al., 2002). The classical explanation for the persistence of fitness‐reducing mutations invokes the antagonistic pleiotropy theory, when the expression of a gene results in multiple competing effects. However, if fecundity compensation has evolved as a response to harmful tumors, the higher fertility in individuals harboring ICMA (e.g., Ohnishil et al., 1997) could be incorrectly interpreted as evidence of antagonistic pleiotropy. To distinguish between the processes that protect ICMA from purging by purifying selection is not just a semantic problem, but an important evolutionary and potentially therapeutic question. Finally, if further studies also support the fact that host life‐history responses can be selected as a state‐dependent response to developing tumors, this will indirectly suggest that cancer constitutes a significant selective pressure shaping the evolution of multicellular organisms. An interesting follow‐up experiment here could be to determine the viability of the eggs produced by cancer and healthy flies, which is the number of individuals that survive from eggs to reproducing adults (realized fecundity). At the moment, there is indeed little evidence available on the consequences of having parents harboring tumors and/or oncogenic mutations on offspring phenotype, in terms of costs and adaptive (nongenetic) transgenerational effects (Ujvari et al., 2016).

## Conflict of Interests

None declared.
